# Detection of Metal-Doped Fluorescent PVC Microplastics in Freshwater Mussels

**DOI:** 10.3390/nano10122363

**Published:** 2020-11-27

**Authors:** Samantha V. Facchetti, Rita La Spina, Francesco Fumagalli, Nicoletta Riccardi, Douglas Gilliland, Jessica Ponti

**Affiliations:** 1 Joint Research Centre (JRC), European Commission, 21027 Ispra, Italy; facchettisamantha@gmail.com (S.V.F.); rita.la-spina@ec.europa.eu (R.L.S.); francesco-Sirio.fumagalli@ec.europa.eu (F.F.); douglas.gilliland@ec.europa.eu (D.G.); 2 Institute of Ecosystem Study, National Research Council (CNR), 28922 Pallanza, Italy; nicoletta.riccardi@irsa.cnr.it

**Keywords:** methods development, metal-doped PVC microplastics, fluorescence, mollusks, mussels, bioaccumulation

## Abstract

The large-scale production of plastic and the resulting release of waste is leading to a huge accumulation of micro-sized particles in the environment that could have an impact on not only aquatic organisms but also on humans. Despite the extensive literature on the subject, there is still an insufficient harmonization of methodologies for the collection and analysis of microplastics (MPs) in complex matrices; especially for high density polymers; such as polyvinyl chloride (PVC), which tend to sink and accumulate in sediments, becoming available to benthonic organisms. In this article, mussels have been chosen as model for microplastic accumulation due to their extensive filtering activity and their wide distribution in both fresh and salt water basins. To facilitate the identification and quantification of microplastics taken up by mussels, novel fluorescent and metal-doped PVC microplastics (PVC-Platinum octaethylporphyrin (PtOEP) MPs in the size range of 100 µm) have been synthesized and characterized. For the analysis of the mussels following exposure, an enzymatic protocol using amylase, lipase, papain, and SDS for organic material digestion and a sucrose-ZnCl_2_ density gradient for the selective separation of ingested microplastics has been developed. The final identification of MPs was performed by fluorescence microscopy. This work can greatly benefit the scientific community by providing a means to study the behavior of PVC MPs, which represent an example of a very relevant yet poorly studied high density polymeric contaminant commonly found in complex environmental matrices.

## 1. Introduction

Although the historical use of synthetic polymers dates back to the early 19th century, in recent decades companies have been producing ever increasing amounts of new synthetic polymers of different chemical compositions, density, sizes and shapes for a wide range of applications, especially in the packaging, construction and automotive industries [1]. In 2018, global production reached about 360 million tons, while in Europe it reached about 62 million tons [2]. In spite of the enormous economic and technical advantages of synthetic polymers (low cost, good resistance to both water and corrosion, easy molding), a large quantity of plastic products on the market are intended for single use (often also referred to as disposable plastics) and thus become plastic waste after a very short life time. A piece of plastic that has remained in our hands for only few seconds can pollute the environment for centuries and eventually contaminate even the most remote corners of the Earth. About 25% of the collected plastic waste ends up in landfills without being recycled or destined for energy recovery [2]. This has created an enormous growing reservoir of plastic lost to the environment, which will age and slowly degrade, resulting in a release of smaller plastic fragments, known as microplastics (MPs, <5 mm [3]). Depending on their origin, they are classified as primary microplastics produced intentionally as additives (microspheres or plastic pellets) [4,5,6] or secondary microplastics generated by fragmentation of larger plastic waste in the environment through natural ageing processes [7,8]. Due to their surface hydrophobicity and large surface area to volume ratio [9], MPs have the potential to adsorb, concentrate, transport and eventually re-release in living organisms and in the soil matrix environmental hydrophobic organic contaminants [10,11,12] and heavy metals such as Cd, Ni, Zn and Pb [13,14]. Because of their small size, often similar to sand grains and planktonic organisms, and their ubiquitous distribution in the aquatic environment [15,16], MPs are available to various organisms in both pelagic and benthic habitats. The monitoring of the presence of MPs in aquatic fauna is currently of great importance as some of these organisms, such as mussels, are consumed daily by humans and can therefore be the main vector of human contamination [17,18]. Due to the well documented advantages of mussels as traditional biological indicators used since the 1960s [19], and mounting evidence of microplastic accumulation, the use of mussels as an indicator of microplastic pollution is recommended [20]. In fact, mussels are the most common benthic species used for studying the fate and toxic effects of microplastics both in the laboratory [20,21] and in the field [22,23,24,25]. Indeed, the evidence of a positive and quantitative correlation of microplastics in mussels and their surrounding waters [23] qualifies it as a suitable tool to detect MPs environmental levels, while laboratory exposure studies demonstrate that mussels can be good model organisms when investigating uptake, accumulation and toxicity of microplastics [26]. Mussels are considered to be suitable model organisms also because of their crucial role in benthopelagic coupling, making them potential vectors towards the higher trophic levels [24]. Although the majority of studies were so far focused on marine habitats [20], the terrestrial and the freshwater environments not only function as sources and transport pathways of plastics to the oceans, but are themselves extensively exposed to anthropogenic litter [27]. To assess the degree of micro- and nano-plastic contamination in freshwater ecosystems, freshwater mussel species are being increasingly used [25,28].

Among the various types of plastic debris found in the environment, polyvinyl chloride (PVC) is one of the most common, given its widespread use as a thermoplastic polymer in numerous applications thanks to its excellent chemical-physical characteristics and its versatility [29,30]. Having a high density (between 1.1 and 1.45 g cm^−3^) [31], this polymer tends to sink and accumulate in sediments, becoming more available to benthonic organisms. The passage of PVC particles via the placenta into fetal circulation has been reported [32]. In addition, PVC often contains a significant proportion of chemical additives such as phthalates, which are able to bind with molecular targets in the body, disrupting hormones [33]. Therefore, it seems to be a highly relevant plastic polymer with regard to dangerous impacts on environmental ecology and possible toxic effects on organisms. To help study the bioaccumulation of PVC in organisms, a procedure has been used to produce easily traceable micro and nanoparticles of the polymer which have been specifically doped with a metal element containing complex [34] or dyes [35,36].

This study aimed to provide a new tool/methodology for the analysis of microplastic bioaccumulation through the: (i) synthesis of new MPs tracers; (ii) evaluation of the detectability of this tracer in complex matrices using different analytical approaches.

To achieve this objective, novel fluorescent and metal-doped PVC-Platinum octaethylporphyrin (PtOEP) MPs have been synthesized, which can be detected by optical methods (fluorescent microscopy). In addition, the entrapment of a metal element within the plastic particles could be a suitable strategy for the indirect determination of the amount of microplastics ingested by the animals via the quantification of Pt internalized by the organism after live exposure, even at low concentrations [34], using trace element analysis methods such as inductively coupled plasma-mass spectrometry [37] or Total Reflection X-ray Fluorescence (TXRF) [38,39]. Freshwater mussels have been used as a complex matrix model organism to study the absorption and bioaccumulation of ingested PVC-PtOEP MPs. These animals are widely used in experiments for MPs evaluation as they can act as bioindicators due to the large volumes of water drawn across the gills when filter feeding [20,40]. In this study, we used wild caught specimens of *Unio elongatulus*, the most abundant native species in Italian freshwater bodies [41]. The bioaccumulation of PVC-PtOEP MPs inside the mussel soft tissues was investigated by exploiting the fluorescent properties of the PtOEP complex within the MPs. In biological matrices, the detection of microplastics requires the extraction and degradation of biogenic matter. Unlike other methods commonly used (treatment with hydrogen peroxide, alkaline/acid digestion, use of Fenton’s reagent [42,43]), enzymatic digestion is a gentle, safe and non-aggressive process to extract MPs in samples containing a high percentage of biological matter, as already reported in the literature [44,45,46]. In this study, the digestion method adopted was based on the use of amylase, lipase and papain followed by an additional purification step using sodium dodecyl sulfate (SDS). This enzymatic-based protocol seems to be effective in breaking down the biological matrix to facilitate the detection and analysis of PVC-PtOEP MPs while preserving the natural chemical and morphological structure of the synthetic polymer. After the enzymatic process, a sucrose-ZnCl_2_ density gradient was used for the final separation of the MPs from residual non-polymeric solids in the digested sample. This high density solution is suitable for the extraction of the common denser polymers (e.g., PVC, PET, PC) that could not be extracted with other solutions as sucrose or NaCl because of the relatively low density (1.2 g cm^−3^) of these solutions [47,48].

## 2. Materials and Methods

### 2.1. Chemicals and Filters

For the synthesis of marked particles, polyvinyl chloride (Mw 43 kDa, PVC), tetrahydrofuran (THF), sodium chloride (NaCl), and platinum octaethylporphyrin (PtOEP) were purchased from Merck (Milan, Italy).

For the enzymatic digestion, α-Amylase from *Bacillus* sp. (A4862-250ML, liquid form), Lipase from *Aspergillus niger* (62301, powder), Papain from *Carica papaya* (10108014001 Roche, Basel, Switzerland, stock concentration 100 mg/10 mL, suspension form) and sodium dodecyl sulphate (SDS, liquid form) were supplied by Sigma-Aldrich (Darmstadt, Germany) such as sucrose and zinc chloride (ZnCl_2_) used for the gradient density separation.

For the filtration step, the membrane filters were made by Whatman^®^, Sigma-Aldrich (Darmstadt, Germany) (filters of cellulose nitrate 12 μm and aluminum oxide (Anodisc) 0.1 μm were used).

### 2.2. Synthesis and Physico-Chemical Characterization of PVC-PtOEP MPs

PVC microplastics were synthesized according to a modification of the protocol described by Zhang et al. [49] A 3% solution of PVC in THF was prepared by dissolution and left to stir overnight at 40 °C before then being further diluted to 1% with THF. Subsequently, 40 mL aliquots of the 1% PVC solution were mixed with 3 mg of PtOEP and left to stir for two hours at room temperature. For the final synthesis of the particulates, the custom experimental set-up shown schematically in Supplementary Materials Figure S1 was used. The two inlets to a T-shaped microchannel junction were connected to a syringe pump (KD Scientific Infusion Only Syringe Pumps) and an HPLC pump. The syringe pump was equipped with a 25 mL glass syringe to deliver the PVC solution, whereas the HPLC pump delivered 20% NaCl aqueous solution. The connective tubing was made of polytetrafluoroethylene (PTFE) with a 0.79 mm, 20 gauge inner diameter. The polymer and 20% NaCl solutions were simultaneously pumped at 100 and 700 μL min^−1^, respectively. Under these conditions, after convergence and mixing of the two streams in the T-junction, droplets were produced and delivered through a stainless steel tube (10 cm, 158 μm i.d.) to a vertically positioned separating funnel containing 1 L of 5% NaCl aqueous solution. A PTFE stopper with a center hole served to connect the stainless steel tube to the separating funnel. After synthesis, the suspension of microparticles was vacuum filtered (Whatman^®^ 25 mm Anodisc inorganic filter membrane, pore size 0.1 μm), rinsed with water and left to dry at room temperature. The marked PVC-PtOEP MPs were then analyzed by fluorescent microscope (Axio Imager 2, Zeiss, Milan, Italy) by illuminating with blue (excitation 475 nm) and green (excitation 555 nm) light.

The volume and number based particle size distributions of the PVC-PtOEP MPs were determined using laser diffraction (LD) (Mastersizer 3000, Malvern, Alfatest, Rome, Italy) and optical microscopy (Axio Imager 2, Zeiss, Milan, Italy), respectively. For the LD measurements, the particulates were dispersed in ethanol before being analysed in the stirred sample cell of the LD instrument operating at 700 rpm. The analysis procedure was repeated four times in order to ensure consistency of the results. The analysis model used assumed that the particle are spherical and the particle refractive index and the particle absorption index for PVC-PtOEP MPs fraction was set to 1.5 and 0.010, respectively. Laser obscurations in the range of 10% and 20% were investigated and the average size was extracted from 10, 50, and 90% of the volume percentile distribution.

The number-based size distribution of the PVC-PtOEP MPs was measured using an optical microscope (Zeiss, Axio Imager 2) in dark field and fluorescent mode. The samples for optical microscopy analysis were prepared on a filter membrane by passing a solution of PVC-PtOEP MPs dispersed in ethanol through 0.1 µm alumina pore membrane (Anopore, Parramatta, Australia) and drying. Multiple images of the PVC-PtOEP MPs on the filter membrane were then acquired and analyzed by the open-source software ImageJ. The collected data were plotted in a histogram to determine the PVC-PtOEP MP mean size distribution by OriginPro 2015. To confirm the effective doping of the particles with the PtOEP compound, the PVC-PtOEP MPs were observed in fluorescent mode.

The presence of the Pt was confirmed by Total Reflectance X-ray Fluorescence (S4 T-STAR, Bruker, Milan, Italy) using Mo (and W) X-ray excitation sources. To prepare samples for TXRF analysis, 1 mg of PVC-PtOEP MPs was suspended in 100 µL of 10% HNO_3_ and spiked with 10 µL of a Ga internal standard (10 mg/L final concentration) and 10 µL of 0.1% Triton X 100 solution. 5 µL was deposited on 30 mm diameter acrylic discs holders and allowed to completely dry prior to TXRF analysis. Samples were prepared in triplicate, air-dried on a hot plate at 45 °C and analyzed immediately.

The chemical characterization of the PVC-PtOEP MPs was carried out by Attenuated Total Reflection Fourier Transform Infrared Spectroscopy (ATR-FTIR, Bruker, Milan, Italy). The spectra were recorded in the absorbance mode in the 4000–600 cm^−1^ region with 2 cm^−1^ resolution and 126 scans. The spectra were acquired and identified by comparing the data to PVC reference spectra in the Opus Bruker software (OPUS 8.5. Version 8.5) analysis.

### 2.3. Experimental Setup and Sampling Procedure

**Figure 1:**
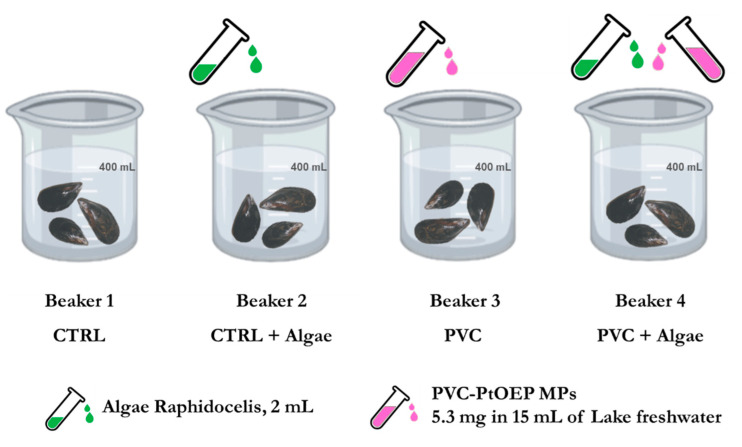
Schematic illustration of the exposure experiments preparation.

Thirty *Unio elongatulus* (Pfeiffer) specimens were collected in Lake Maggiore (VA) Italy at 5 m depth. After sampling and cleaning the shells of the peryphitic film, the mussels were kept in a flow-through aquarium in filtered (0.45 µm) lake water for 48 h to allow complete gut clearance. Before the experiment, 12 randomly selected mussels were acclimatized in the dark for 3 days in a oscillation incubator at 15 °C and 50 rpm in a large maintenance glass beaker containing ca. 4 L of filtered (0.45 µm) lake water. Mussels were then fed once with a *Raphidocelis* sp. culture and the water was aerated twice for 3 h during this pre-experiment step. The successive exposure experiments were conducted with mussels randomly assigned to one of following treatments (Figure 1): two controls (Beaker 1 with no additives and Beaker 2 with 2 mL of algae), 5.3 mg of PVC-PtOEP MPs (Beaker 3) and 5.3 mg PVC-PtOEP MPs + 2 mL algae (Beaker 4). PVC-PtOEP MPs were previously dispersed in 15 mL of freshwater and sonicated for 2 min. In each of the experiments, 400 mL of filtered lake water (Nalgene^®^ filter, pore size 0.45 µm, Sigma-Aldrich, Milan, Italy) and three comparably sized mussels randomly selected from the main mussel maintenance tank had been previously added. Considering the final volumes in Beaker 3 (415 mL) and Beaker 4 (417 mL), the final exposure concentrations to PVC-PtOEP MPs were 12.77 µg/mL and 12.70 µg/mL, respectively. Animals were exposed for 4 h at 15 °C in the dark (50 rpm). The 4 h exposure time was chosen to increase the probability of particle uptake, thus matching the methodological aim of the experiment. The time selection was based on the average clearance and excretion rates measured for *Unio elongatulus* specimens from the same Lake Maggiore population under the same experimental conditions. The values (N. Riccardi, unpublished data) were used to define the time needed to ensure the filtration of the whole water volume without causing an excessive accumulation of mussel byproducts in the treatments.

Mortality of exposed mussels was zero during the experiments. After exposure, mussels were removed from the exposure bucket and thoroughly rinsed with filtered Milli-Q water. After mussels removal, feces/pseudofeces were collected from the bottom of the beaker with a pipette and preserved for microscope observation. Two mussels from each treatment were frozen at −20°C (for further enzymatic digestion), while one was sacrificed immediately and dissected to evaluate the bioaccumulation of microplastics in the mantle, gills, digestive gland, and gonad. Each dissected part was placed on a glass slide and screened under fluorescent microscope by irradiation under blue (excitation 475 nm) and green (excitation 555 nm) light.

### 2.4. Enzymatic Purification and Digestion Protocol

After careful qualitative evaluation of the distribution of microplastics in the different animals’ organs and in the presence or absence of algae, one mussel per treatment was enzymatically digested (whole body) to reduce the biological matrix. The final optimized digestive protocol includes a sequence of three purification steps through the action of technical enzymes (amylase, lipase, and protease) and, as a last step, the addition of SDS (Figure S2).

In order to prevent sample (cross) contamination, all laboratory equipment and materials were thoroughly rinsed with prefiltered (0.22 μm) deionized Milli-Q water before all working steps and, wherever possible, equipment made of plastic was replaced with glass or metal components. All enzymes and solutions used were passed through a 0.22 μm pore filter before use.

The enzymatic digestion was carried out at 50 °C; this temperature was selected as it is within the range of action of all the enzymes and is unlikely to affect plastics. Before starting with the digestion process, mussels were removed from the freezer and allowed to defrost for one hour. They were then shelled, slightly drained, weighed (an average weight of 4 g was calculated) and placed in a 30 mL glass vial for digestion (1 sample/vial). In order to homogenize the sample as much as possible, it was cut with scissors and bath sonicated for about 15–20 min.

The first enzymatic purification step was conducted with 4 mL of α-Amylase from *Bacillus sp* (1 mL/1 g of sample). Once the solution was added, the vial was placed in an oven at 50 °C for 3 h under stirring. Subsequently, 0.5 mg Lipase from *Aspergillus niger* was added per gram of sample. Before use, the enzyme was completely dissolved in 1 mL of Milli-Q water (2 mg/mL) and then the solution was added to the sample for the digestion. Incubation was carried out for 3 h at 50 °C under stirring.

To perform the enzymatic digestion of proteins, papain from *Carica papaia* was selected. Prior to use, 300 µL of papain stock solution, corresponding to a final concentration of 0.5 mg/mL, must be activated for 30 min at 50 °C within 1 mL of buffer consisting of: 11 µL of EDTA (stock solution 100 mM), 30 mg L-cysteine-HCl (final concentration of 0.5% m/V) and 0.689 mL of Milli-Q water. Once activated, the enzyme solution was mixed with the sample and the digestion allowed to proceed overnight at 50 °C under stirring. As the last purification step, 1 mL of 2% SDS was added to the sample and allowed to react for 1.5 h at 50 °C under stirring. After digestion, all the enzymes were deactivated by heating up to T ≥ 80 °C for 15–20 min, the digested sample was cooled to room temperature, and 400 µL of Penicillin-Streptomycin (antimicrobial agent) was added. Samples were then stored at 4 °C for further analysis.

Mussel samples were analyzed by optical microscopy to evaluate if the enzymatic degradation was successful; 5 µL of digested sample after papain treatment and SDS treatment were spotted onto glass slides, protected with a coverslip, and examined with dark field optical microscopy (20× objective). From the resulting images, the dimension and quantity of particulates remaining in the samples was investigated.

### 2.5. Sucrose-ZnCl_2_ Density Gradient Centrifugation

The digested sample after enzymatic treatment still contains both inorganic (e.g., sand and diatom frustules) and organic particulates of different density and size. To separate all unwanted material from the PVC-PtOEP MPs, a density gradient purification step was selected. Eight solutions (Figure S3) based on variable combination of sucrose and ZnCl_2_ were prepared as shown in Table 1.

With a sucrose gradient, a maximum concentration of 1.3 g/cm^3^ can be achieved, while mixing sucrose with ZnCl_2_, which is available at a low cost and has a rather high water solubility, it is easy to obtain 2.0 g/cm^3^. This allows an excellent fractionation of denser inorganic material from organic matter.

When necessary, heating and stirring were applied to ensure that the salt and sucrose had been completely solubilized. To make an approximate density calculation, a portion of each solution was transferred into a 1 mL Eppendorf and weighed. In this way, considering a final volume of 1 mL, the density was estimated using the relationship: d (g/mL) = m (g)/V (mL).

The gradient was then prepared by layering 5 mL of each solution in a 50 mL polypropylene centrifugation tube. The digested sample was loaded on top of the specific gravity solutions and the gradient separation was performed through a 10 min centrifugation (3992 rcf). To find out where the PVC-PtOEP MPs were positioned in the gradient, each fraction was manually separated and analyzed by fluorescent microscope using illumination with blue (excitation 475 nm) and green (excitation 555 nm) light. Once the correct fraction was identified, the ingested micro PVC-PtOEP particles were recovered by vacuum filtration (Whatman^®^ Cellulose Nitrate Membrane Filter 12 µm) and washed with approximately 30–40 mL of Milli-Q water in order to clean the plastic particles from the salt-sucrose solution and matrix residues. Next, the filter was placed into a clean petri dish with a cover for further analysis.2.6. Identification of Ingested PVC-PtOEP MPs by Fluorescent Microscopy.

Once the PVC-PtOEP MPs were isolated with the gradient and filtered, the filter was analyzed with the fluorescence microscope) under illumination by blue (excitation 475 nm) and green (excitation 555 nm) light to perform particle identification.

## 3. Results

### 3.1. Synthesis and Physico-Chemical Characterization of PVC-PtOEP MPs

**Figure 2:**
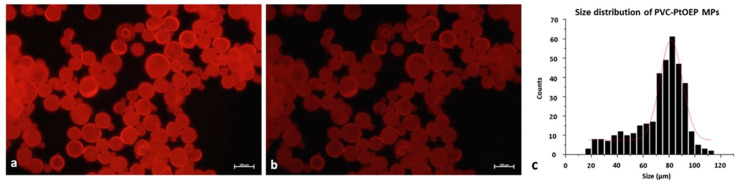
Fluorescent images of PVC-PtOEP MPs irradiate with (**a**) blue and (**b**) green light in the same conditions of illumination and exposure time. Scale bar 100µm. (**c**) represents the size (µm) distribution of PVC-PtOEP MPs.

The characterization of PVC-PtOEP MPs was carried out by laser diffraction and the size and particle size distribution, extracted at 10, 50, and 90% of the volume percentile distribution, was 62 ± 1, 120 ± 5, and 380 ± 80 µm, respectively. In addition, Figure 2 shows the optical images of PVC-PtOEP MPs in fluorescent under the blue and the green lights. The images were then analyzed by the free software ImageJ to plot the size distribution (Figure 2c) resulting in a mean size of 80 µm.

The chemical characterization of the PVC-PtOEP MPs was carried out by ATR-FTIR (Figure S4), confirming the PVC composition of the particles, without any interference from PtOEP dye.

**Figure 3:**
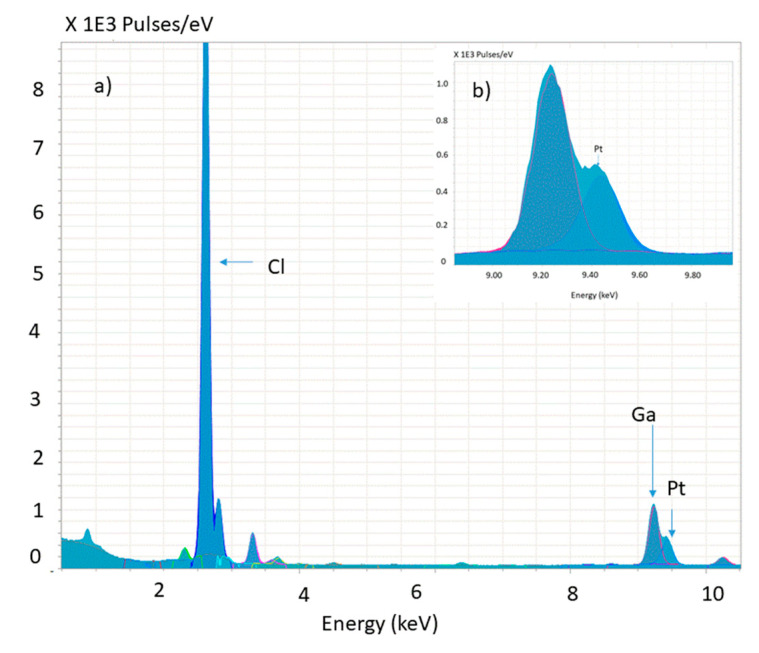
(**a**) TXRF analysis of the PVC-PtOEP MPs confirming the presence of Pt into the particles. Ga is present as the internal standard. (**b**) inset of the peak related to the Pt.

A qualitative analysis was carried out by TXRF to detect the presence of Pt into the particles, which is confirmed in Figure 3.

### 3.2. Experimental Setup and Sampling Procedure

#### 3.2.1. Mussel Dissection

The exposure of an organism as a whole (in vivo study) is useful to assess some biological effects of microplastics such as bioaccumulation. In Figure S5 the four parts (mantle, gills, digestive gland, gonad) in which the animals were dissected are shown. Observation under a fluorescence microscope revealed where the PVC-PtOEP particles were located in tissues after ingestion by the mussels.

In both the controls, no PVC-PtOEP MPs were observed, while they were found in both exposure treatments, with and without algae addition. Only a few particles were present on the mantles and on the gills. Conversely, a high concentration of particles was found in both gonad and digestive glands.

Many small sized PVC-PtOEP MPs surrounded by mucus were found in the feces and pseudofeces of the animals after exposure.

#### 3.2.2. Enzymatic Purification

##### Efficiency of the Digestion Protocol

**Figure 4:**
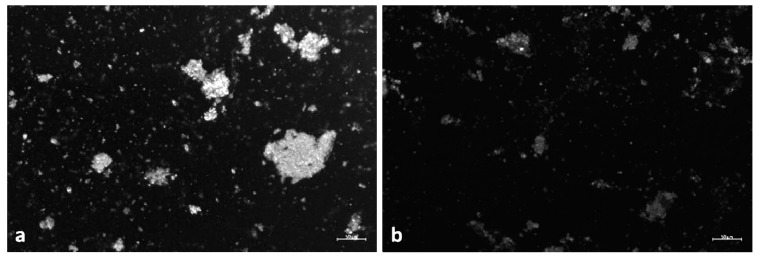
Digestion efficiency evaluation after (**a**) amylase-lipase-papain digestion and (**b**) after additional SDS purification step. Scale bar 50 µm.

The enzymatic-based digestion protocol used in this experiment was qualitatively proved to be efficient in reducing the biological materials present within the sample. In fact, as shown in Figure 4, the sample matrix was largely eliminated after the sequential enzymatic purification and SDS purification steps. Furthermore, experiments previously conducted by our group have shown that the combination of amylase, lipase, and papain results in a better purification than digestion with papain alone (Figure S6). From the microscopic picture, a decrease in the amount of residual material visible after treatment with SDS can be observed, which confirms the ability of SDS, an anionic surfactant, to macerate lipids, planktonic organisms, and plant residues.

##### Sucrose-ZnCl_2_ Density Gradient Centrifugation

**Figure 5:**
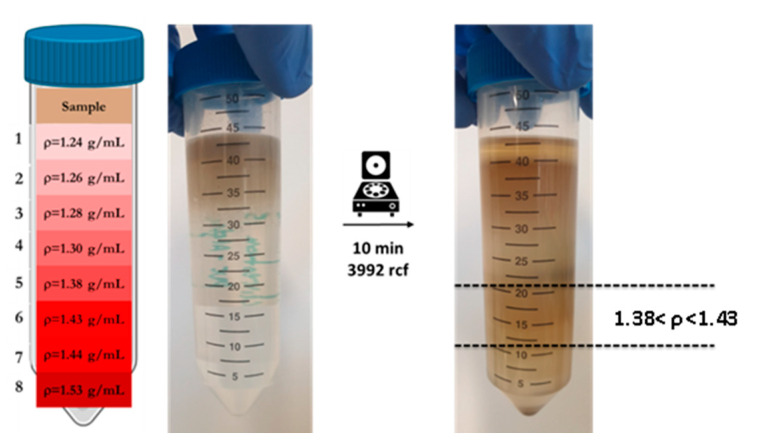
Separation, according to density, of the sample digested through a sucrose-ZnCl_2_ gradient by centrifugation.

Figure 5 shows the effectiveness of the separation through the gradient described above. The layer in which the PVC-PtOEP MP particles are internalized by the animal found by fluorescent microscopy analysis is highlighted with the black dotted lines.

### 3.3. Identification of Ingested PVC-PtOEP MPs

#### Fluorescent Microscopy Identification

**Figure 6:**
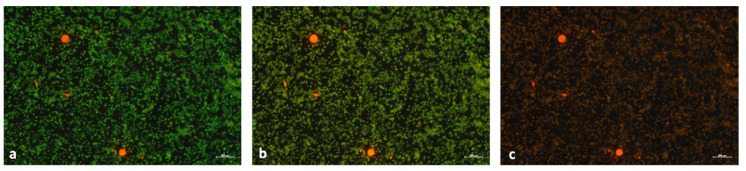
Images of the filter taken with the fluorescence microscope under blue light (**a**), blue and green lights (**b**) and green light (**c**). The red dots represent the PVC-PtOEP MPs ingested from the mussel and extracted with the density gradient. Scale bar 200 µm.

As shown in Figure 6, PVC-PtOEP MPs extracted from the mussel matrix after enzymatic digestion and density gradient separation are easily detected on the filter by fluorescence microscopy analysis under both blue (a) and green (c) light. In the central photographic representation (b) the sample was irradiated simultaneously with blue and green light, and the combination of the two generates a bright red color that allows a better identification of the PVC-PtOEP MPs, especially for the smaller particles.

## 4. Discussion

In this article, fluorescent and metal-doped PVC microparticles have been synthesized. Their potential for use in both bioaccumulation studies and as a means to localize microplastics in complex matrices offers a great advantage for researchers attempting to understand the behavior of PVC MPs, a highly relevant yet less studied polymeric contaminant in environmental samples. Polyvinyl chloride is in fact one of the main synthetic polymers produced for various applications and are therefore certainly present in plastic debris and are especially localized on seabed and freshwater basins due to its high density. The labeling of the MPs synthesized here with a novel, dual function marker which is both a fluorescent dye and an elemental tracer permits the option of using both optical and elementary analysis techniques to detect the presence and/or accumulation of MPs in tissues or complex organisms. With regard to their fluorescence properties, an important advantage of the PVC-PtOEP MPs discussed here is that their fluorescent is not limited to a single excitation band (like most MPs used in the literature), but two (blue and green light, in Figure 2). This makes them ideal as a material for identification studies of microplastics in natural auto-fluorescent samples as they are perfectly distinguishable from any other endogenous or xenobiotic particles (Figure 6) often present in the matrix (such as algae). Furthermore, the presence of an encapsulated metal-organic compound containing a rare element such Pt as a tracer presents interesting possibilities for using the element specific to analytical methods (ICP-MS) for tracking/quantifying particles which may difficult to analyze due to low concentrations, very small size, or interference from a high natural background.

From the characterization carried out here of the MPs in PVC-PtOEP, it has been shown that PtOEP is a labeling agent suitable for following the bioaccumulation of plastic in organisms. In addition, the release of PtOEP and Pt during analysis can be largely excluded, because the dye is insoluble in aqueous media and experimental observations have shown no indication of any movement of residual fluorescence into the media and tissue when compared to control samples. From the assessment of bioaccumulation within the organism, qualitative analysis shows that the plastic particles observed on the mantle appear larger than those observed in other tissues, indicating that the particle size is a factor which is relevant to localized accumulation rates. In general, the smaller the microplastics, the greater the absorption and translocation. This could suggest that PVC-PtOEP MPs were drawn through the inhalant siphon and filtered via the gill. On the gill, filamentous cilia capture plastic particles and rapidly transport them to the ventral groove and then on to the labial palps, where cilia sort them for ingestion in the digestive tract. More MPs of PVC-PtOEP were observed in the tissues of the algae-fed mussels (Beaker 4) than in the starving mussels (Beaker 3), confirming the effect of algae uptake. This may be due both to a previous interaction between MPs and algae before being filtered by the animal and to the presence of food particles, which stimulate the filtering activity of the mussels [50]. Although the experiment was not designed for a quantitative assessment of uptake and accumulation, the difference between the number of particles detected in starved vs fed organisms has important methodological and interpretative implications. The common practice of carrying out short-term eco-toxicological experiments with starved animals can severely hinder the extrapolation of the results to natural conditions. The uptake in the field can reflect the response of organisms to environmental conditions (e.g., quantity and quality of the food available) besides the quantity, quality, and distribution of the micro-nano plastic debris.

The enzymatic sequence developed for the purification of the organic matrix involved the use of the three enzymes-amylase, lipase, and protease (Figures S2 and S6). In particular, it was decided to start the digestion protocol with amylase considering its abundance in human saliva, where the mechanical process of digestion begins. In addition, papain was added after amylase and lipase because, being a protease, it could destroy the other proteins and therefore the previous enzymes. The choice of adding SDS as a last step is due to its possible ability to denature protease [51]. Therefore, this protocol seems to be a good compromise to avoid multi-centrifugation and enzyme deactivation steps.

Before digestion, the specimens were frozen at −20 °C and, after thawing, ultrasonicated for a short time in order to weaken the tissues, thus facilitating the digestion; no evidence of morphological changes to the particles was observed as result of these steps. During the entire digestion process, no other ultrasonication steps were done to avoid any possible degradation of the plastics or formation of artificial secondary microplastics.

The amylase enzyme used has the advantage that it is supplied in a liquid form that does not need to be buffered and it is easier to handle in comparison to the powdered enzymes. Papain was studied for the digestion of protein species. Despite being redox sensitive and requiring activation, it is cheaper than proteinase K, often used by other authors, and has a high specificity for peptide bonds and an adequate level of enzymatic activity [52].

After enzymatic treatment, the sample still contained an incompletely digested fraction of organic matrix, algae, and very fine inorganic sandy particulates which interfered with the identification of ingested MPs. It was therefore necessary to exploit the different densities of the various components in order to further purify the sample and allow greater separation of the MPs. This was done by density gradient centrifugation using a series of sucrose and sucrose-ZnCl_2_ solutions. It was found that by adding ZnCl_2_ to the lower layers of a sucrose gradient (Figure 5), it was possible to obtain solutions with an optimal density for separating the plastic polymer used in this experiment from both higher density minerals and lower density organic residues. This gradient has also been found to be very useful for the specific extraction of the labeled plastic microbeads in complex matrices where other fluorescent components, such as algae, may be present.

In conclusion, the combination of the enzymatic protocol reported here, the density gradient developed and the effective fluorescent detection made it possible to successfully extract and identify the PVC-PtOEP microplastics internalized into the complex matrix of the fresh water mussels which are an environmentally relevant accumulator of microplastics. This method can be extended to other type of plastics found in the environment.

## Figures and Tables

**Table 1:** Quantities of salt (ZnCl_2_) and sucrose in 5 mL of Milli-Q water used for the density gradient solution preparation. The calculated densities are reported.

	Amount of Solute in 5 mL Water		
Solution	ZnCl_2_ (g)	Sucrose (g)	Density (g/mL)
1	-	5.50	1.24
2	-	6.50	1.26
3	-	7.50	1.28
4	-	9.75	1.30
5	1.50	9.75	1.38
6	2.00	9.75	1.43
7	2.50	9.75	1.44
8	5.50	9.75	1.53

## Supplementary Materials

The following are available online at https://www.mdpi.com/2079-4991/10/12/2363/s1, Figure S1: Schematic representation of the microsphere fabrication device, Figure S2: Flowchart of the optimized enzymatic digestion protocol used, Figure S3: Solutions for the density gradient, Figure S4: FT-IR of PVC-PtOEP MPs, Figure S5: Soft tissues after dissection from mussels to assess PVC-PtOEP MPs bioaccumulation, Figure S6: Images of matrix obtained by two different enzymatic digestion protocols.
