# Supplementary material for: Detection of Metal-Doped Fluorescent PVC Microplastics in Freshwater Mussels
Source: Nanomaterials (Basel). 2020 Nov 27;10(12):2363. doi: 10.3390/nano10122363 (PMC7759941; doi:10.3390/nano10122363)
Supplement: Supplementary file 1 [file nanomaterials-10-02363-s001.pdf]

Detection of metal-doped fluorescent PVC microplastics in freshwater mussels

Samantha V. Facchetti [1], Rita La Spina [1], Francesco Fumagalli [1], Nicoletta Riccardi [2], Douglas Gilliland [1] and Jessica Ponti [1,*]

[1] Joint Research Centre (JRC), European Commission, 21027 Ispra, Italy; facchettisamantha@gmail.com (S.V.F.); rita.la-spina@ec.europa.eu (R.L.S.); francesco-Sirio.fumagalli@ec.europa.eu (F.F.); douglas.gilliland@ec.europa.eu (D.G.)

[2] Institute of Ecosystem Study, National Research Council (CNR), 28922 Pallanza, Italy; nicoletta.riccardi@irsa.cnr.it

* Correspondence: jessica.ponti@ec.europa.eu; Tel.: +39-0332-785793

SUPPORTING INFORMATION

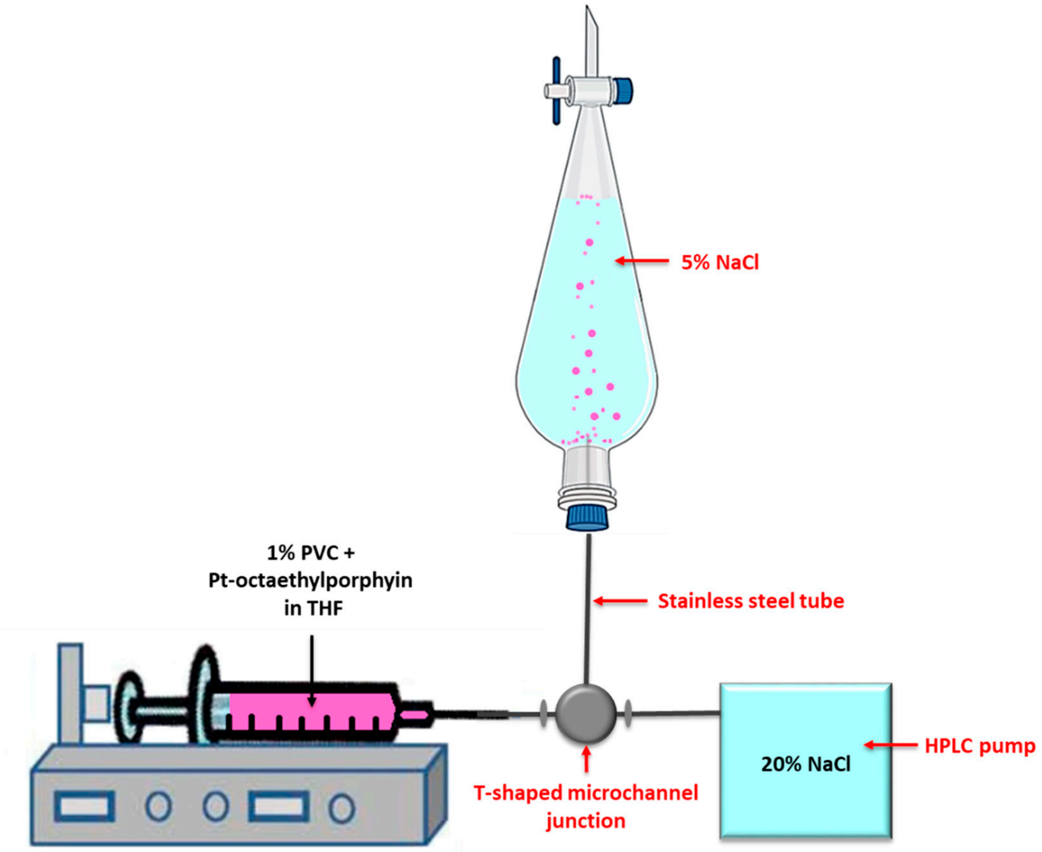

Figure S1. Schematic representation of the microsphere fabrication device.

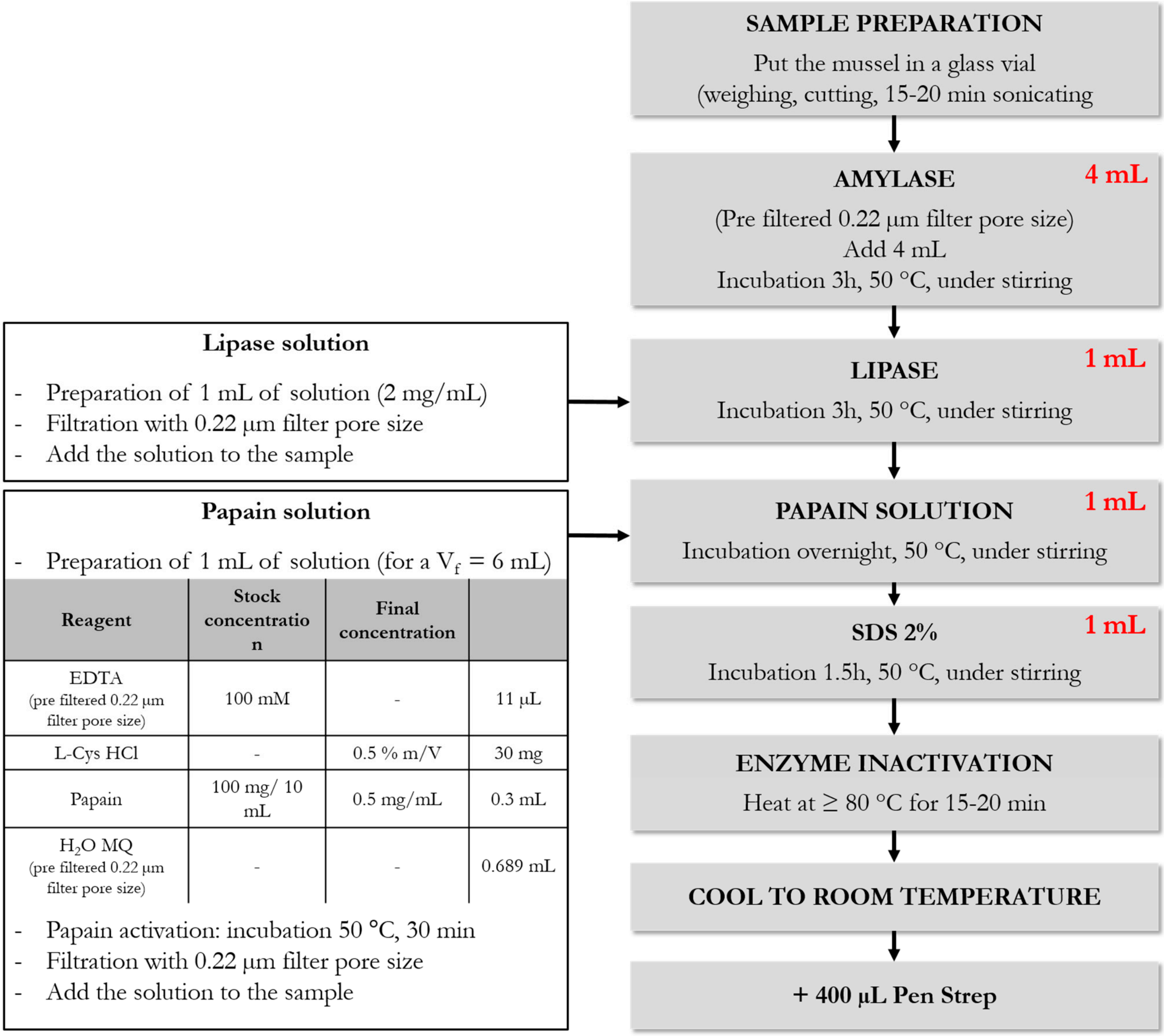

Figure S2. Flowchart of the optimized enzymatic digestion protocol used.

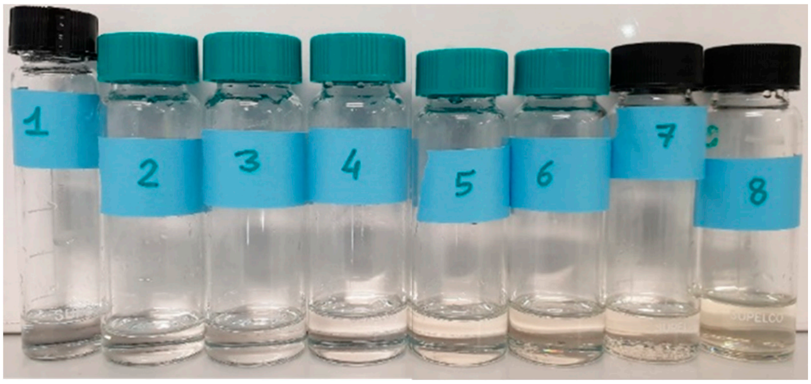

Figure S3. Solutions for the density gradient. From the image it is possible to note a change in color as the salt content increases.

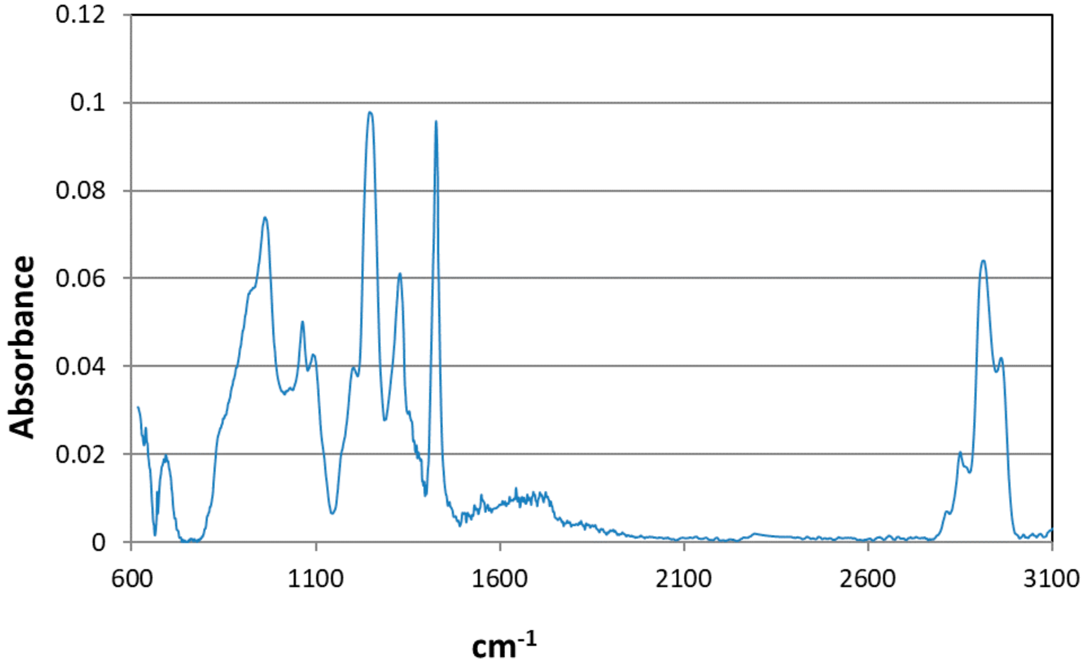

Figure S4. FT-IR of PVC-PtOEP MPs. Spectral identification was done using Opus analysis software, Bruker.

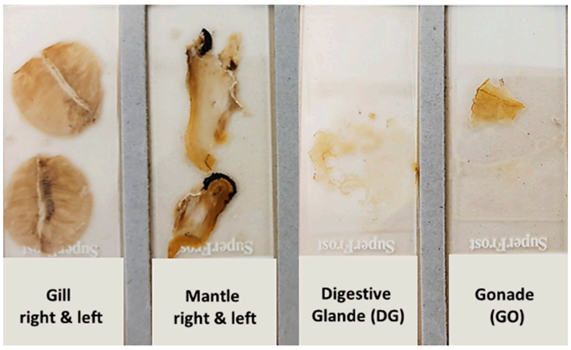

Figure S5. Soft tissues after dissection from mussels to assess PVC-PtOEP MPs bioaccumulation.

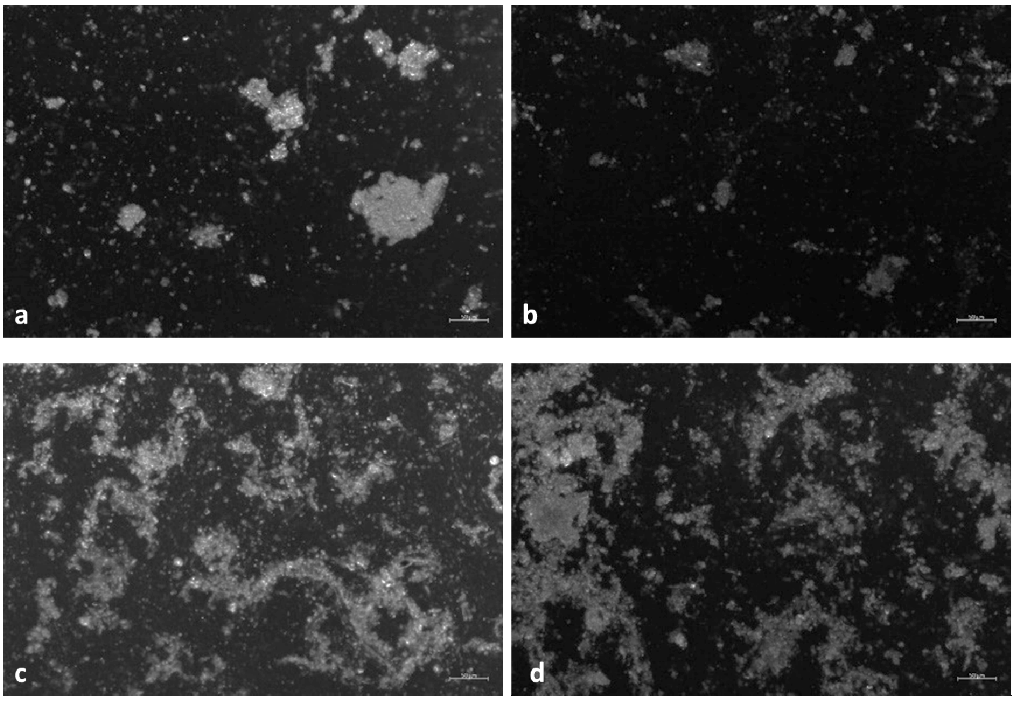

Figure S6. Evaluation of digestion efficiency between two different enzymatic digestion protocols. The two upper images refer to the protocol used in this article with (a) being the matrix after amylase, lipase, papain digestion alone while (b) includes also a final addition of SDS. The two lower images below refer to an alternative, simpler papain only protocol tested by our research group. Figure (c) shows matrix after only papain digestion while figure (d) is the papain digest after SDS addition. This illustrates how the combination of several enzymes before the addition of SDS leads to a greater evident purification of the matrix.
